# Taxonomic Biomarkers of Gut Microbiota with Potential Clinical Utility in Mexican Adults with Obesity and Depressive and Anxiety Symptoms

**DOI:** 10.3390/microorganisms13081828

**Published:** 2025-08-05

**Authors:** María Alejandra Samudio-Cruz, Daniel Cerqueda-García, Elizabeth Cabrera-Ruiz, Alexandra Luna-Angulo, Samuel Canizales-Quinteros, Carlos Landa-Solis, Gabriela Angélica Martínez-Nava, Paul Carrillo-Mora, Edgar Rangel-López, Juan Ríos-Martínez, Blanca López-Contreras, Jesús Fernando Valencia-León, Laura Sánchez-Chapul

**Affiliations:** 1 División de Neurociencias Clínicas, Instituto Nacional de Rehabilitación “Luis Guillermo Ibarra Ibarra”, Mexico City 14389, Mexico; psic.alejandra.samudio@gmail.com (M.A.S.-C.); neuropolaco@yahoo.com.mx (P.C.-M.); 2 Clúster Científico y Tecnológico BioMimic^®^, Red de Manejo Biorracional de Plagas y Vectores, Instituto de Ecología, A.C.—INECOL, Xalapa 91070, Mexico; daniel.cerqueda@inecol.mx; 3 División de Neurociencias Básicas, Instituto Nacional de Rehabilitación “Luis Guillermo Ibarra Ibarra”, Mexico City 14389, Mexico; elicabreraruiz@gmail.com; 4 Laboratorio de Enfermedades Neuromusculares, División de Neurociencias Clínicas, Instituto Nacional de Rehabilitación “Luis Guillermo Ibarra Ibarra”, Mexico City 14389, Mexico; lunangulo@gmail.com; 5 Unidad de Genómica de Poblaciones Aplicada a la Salud, Facultad de Química, Universidad Nacional Autónoma de México (UNAM)/Instituto Nacional de Medicina Genómica (INMEGEN), Mexico City 14610, Mexico; cani@unam.mx (S.C.-Q.); blopez@inmegen.gob.mx (B.L.-C.); 6 Unidad de Ingeniería de Tejidos, Terapia Celular y Medicina Regenerativa, Instituto Nacional de Rehabilitación “Luis Guillermo Ibarra Ibarra”, Mexico City 14389, Mexico; cls_73@hotmail.com; 7 Laboratorio de Gerociencias, Instituto Nacional de Rehabilitación “Luis Guillermo Ibarra Ibarra”, Mexico City 14389, Mexico; ameria.justice@gmail.com; 8 Laboratorio de Reprogramación Celular, Instituto Nacional de Neurología y Neurocirugía “Manuel Velasco Suárez”, Mexico City 14269, Mexico; raledg@hotmail.com; 9 Instituto de Investigación en Ciencias de la Salud, Secretaria de Marina, Mexico City 04830, Mexico; juan_rios_mtz@yahoo.com.mx; 10 Dirección General Adjunta de Sanidad Naval, Secretaria de Marina, Mexico City 04830, Mexico; jesus_ferval@hotmail.com

**Keywords:** depression, anxiety, obesity, *Escherichia coli*, gut–brain axis, gut microbiota, Mexican population

## Abstract

While the gut microbiota of obese children in Mexico has been studied, its relationship with depressive and anxiety symptoms in obese adults remains unexplored. The aim of this study was to describe the gut microbiota profile of Mexican adults with obesity and its association with depression and anxiety. We sequenced the V3-V4 region of the 16S rRNA gene from stool samples of obese adults categorized into four groups: control (OCG), with depressive symptoms (OD), with anxiety symptoms (OAx), or with both (ODAx). Alpha diversity was assessed using *t*-tests, beta diversity was assessed with PERMANOVA, and taxonomic differences was assessed with LEfSe. Associations between bacterial genera and clinical variables were analyzed using the Maaslin2 library. Bacteroidota was the most prevalent phylum, and *Prevotella* was the dominant enterotype across all groups. Although overall diversity did not differ significantly, 30 distinct taxonomic biomarkers were identified among groups as follows: 4 in OCG (Firmicutes), 5 in OD (Firmicutes, Bacteroidota), 13 in OAx (Firmicutes, Bacteroidetes, Fusobacteroidota, Proteobacteria), and 8 in ODAx (Firmicutes). This is the first study to identify distinct gut microbiota profiles in obese Mexican adults with depressive and anxiety symptoms. These findings suggest important microbial biomarkers for improving the diagnosis and treatment of mental health conditions in obesity.

## 1. Introduction

Depression and anxiety are the most common mental disorders worldwide, with the percentage of adults suffering from them standing at 5% and 4%, respectively [1]. The Mexican population has a prevalence of 15.4% for depression and 31.3% for anxiety in adults, which are among the highest in the world [2]. Both mental disorders occur as comorbidities in many diseases, such as obesity [3], which increases the incidence of these disorders [4,5,6], and a high body mass index (BMI) is considered a risk factor, as it predicts a chronic course of depressive and anxiety symptoms [7,8]. Depression has a bidirectional relationship with obesity. People with depression have a 50% risk of developing obesity, and people with obesity have a higher risk of developing depressive symptoms [8]. Further, obesity increases the likelihood of suffering from an anxiety disorder or anxiety symptoms by 30% and 40%, respectively [7].

Obesity affects 16% of adults worldwide [9], and Mexico is one of the countries with the highest prevalence of obesity (36.9%) in adults over 20 years of age [10]. Obesity is caused by an imbalance between caloric intake and caloric expenditure, defined as a BMI > 30 kg/m^2^ [9]. Poor eating habits are associated with changes in the gut microbiota and metabolite production that affect both health and behavior [8,11]. In mice whose microbiota was decimated by antibiotics, a correlation between mood and microbiota was observed. After the transference of feces from donor mice fed a high-fat diet, these mice showed more anxiety-like behavior compared to mice that received the microbiota from normally fed individuals [12]. Preclinical studies in which the fecal microbiota of patients with depression was transplanted to germ-free mice showed decreased motility in various tests, suggesting that such transplantation induces depression-like behavior in recipient mice [13]. Clinical studies have investigated the effects of transplanting the fecal microbiome from healthy donors on patients with depression, using the Hamilton Depression Rating Scale (HAM-D) to assess outcomes. These studies showed a significant short-term improvement in depressive symptoms, although the long-term effects were less consistent [13]. Consequently, the microbiota–gut–brain axis is increasingly recognized as a bidirectional communication system linking brain and gastrointestinal functions [8,11,14].

Depression, anxiety, and obesity are disorders that are currently the subject of intensive research. However, further studies are needed to characterize the structure and metabolic profile of the microbiota to better understand the modulation of the microbiota–gut–brain axis in order to propose new forms of early diagnosis and possible treatments focused on the composition of the microbiota in understudied populations such as those in Mexico. Metabolic characteristics and the taxonomic profile of the gut microbiota related to obesity have been reported for this population in adult women with obesity and obesity plus metabolic syndrome [15], and in adults with different stages of type 2 diabetes mellitus [16], but none of these studies in Mexico have described the situation of depressive and anxiety symptoms in obese individuals. Therefore, the aim of this work was to describe the gut microbiota profile of Mexican adults with obesity and its association with depression and anxiety.

## 2. Materials and Methods

### 2.1. Participants

One hundred and six individuals of both sexes aged 24–58 years from the Mexican Navy, with diagnosed obesity (BMI ≥ 30 kg/m^2^) and without neurological or psychomotor disorders, were included in this study. Navy personnel are a recruited population that is exposed to chronic stress—irregular duty schedules, hostile environments, sleep disturbances, and physical demands—all of which can greatly affect the gut microbiota. This makes them an ideal homogeneous model to study how chronic stress affects gut health, psychological well-being, and metabolism [17]. The Beck Depression Inventory (BDI) and Anxiety Inventory (BAI) were used to determine the presence and severity of these symptoms. The BAI and BDI are 21-item self-report inventories that measure characteristic attitudes and symptoms of anxiety and depression; a higher score indicates more severe symptoms. They were applied individually by a Mexican Navy psychologist in each cabin both before the stool and blood samples were taken and three months later. Taking into account the presence or absence of depressive and anxiety symptoms according to the cut-off points of the BDI (10 points) and the BAI (6 points), participants were divided into two main study groups: a control group (OCG) consisting of obese participants without symptoms of depression or anxiety (n = 79), and an obese group representing all included participants with depressive and anxiety symptoms (OwS) (n = 27). The OwS group represented the 3 groups, which included an obese group with depressive symptoms (OD) (n = 7); an obese group with anxiety symptoms (OAx) (n = 8), and an obese group with both depressive and anxiety symptoms (ODAx) (n = 12). Participants with diarrhea or who had taken antibiotics within 3 months before the start of the study, as well as laxatives or probiotics before the stool sampling, were excluded. Weight, height, and body composition (percentage of fat mass, lean mass, and muscle mass) were determined using InBody analysis (InBody 270), for which all participants were asked to wear shorts and not to exercise or eat for 3–4 h before the test. BMI (kg/m^2^) was determined as the result of the ratio of weight (kg) to height squared (m^2^), with obesity defined as a BMI of ≥30 kg/m^2^. All measurements were performed by a nutritionist with ISAK (International Society for the Advancement of Kinanthropometry) level 2 accreditation from the Mexican Navy on the same day and in the same session to avoid technical measurement errors. Intakes of energy (kcal/day), carbohydrates (g/day), proteins (g/day), and fats (g/day) were determined using a food frequency questionnaire [18]. The information on physical activity and medication intake was taken from the clinical assessment interview; unfortunately, the level of physical activity was not measured. Exercise was considered occasional as it occurred 1–3 times per week and consisted of cycling, soccer (every 15 days), walking, swimming, basketball, Zumba, volleyball, running, boxing, baseball, frontenis. Sixty participants (40%) were taking the following medications: diclofenac, captopril, metformin, enalapril, ketoprofen, naproxen, salbutamol, propranolol, losartan, pregabalin, carbamazepine, folic acid, and levothyroxine. This study was approved by the Research and Ethics Committee of the National Institute of Rehabilitation “Luis Guillermo Ibarra” (CONBIO-ETI-CA-09-CEI-03120171207). All Navy personnel were informed of the benefits and risks of the study before signing an institutional informed consent form to enable them to participate in the study.

### 2.2. Laboratory Data Collection

Blood samples were collected by venipuncture after 12 h fast (Vacutainer, Becton, Dickinson and Company, Franklin Lakes, NJ, USA). The serum was immediately separated by centrifugation and stored at −80 °C until use. Serum biochemistry, lipids, and liver values were assessed at the Naval Medical Center Clinical Pathology Laboratory.

### 2.3. Stool Sample Collection and DNA Extraction

Participants were given a kit containing a clean, dry, and sterile plastic container and a scoop. They were instructed to collect the stool with the scoop and place a small amount in the container. Participants were asked to collect the first sample of the morning, to avoid contamination with urine or toilet water, to close the container tightly and to label it clearly after collection. All samples were kept refrigerated and transported to the laboratory in a container on ice. Upon arrival at the laboratory, 4 aliquots of 180–200 mg each were prepared and stored at −80 °C until processing. Total bacterial DNA was extracted from 200 mg of feces using a commercial kit (QIAamp DNA Stool Mini Kit-Qiagen, Hilden, Germany) according to the manufacturer’s instructions. The final elution volume was 100 μL and the sample was stored at −20 °C until further analysis. DNA concentration and purity were determined via fluorescence using a ThermoFisher^®^ Qubit fluorometer, and spectrometry was determined using the NanoDrop One instrument (ThermoFisher Scientific, Waltham, MA, USA), respectively.

### 2.4. Gut Microbiota Analysis

Microbiota analysis was performed by sequencing the hypervariable region V3-V4 of the 16S ribosomal gene using the following primers: 341F (5′-CCTACGGGNGGCWGCAG-3′) and 805R (5′-GACTACHVGGTATCTAATCC-3′). For the first PCR, we took 2.5 µL of genomic DNA (5 ng/µL) per sample, 0.5 µL of each primer, and 12.5 µL of 2x KAPPA HiFi HotStart ReadyMix in a total volume of 25 µL to realize 25 cycles of 95 °C for 30 s, 55 °C for 30 s, 72 °C for 30 s, and a final extension at 72 °C for 5 min. For quality control of the amplification of the V3–V4 hypervariable region, we then performed electrophoresis of the amplicons on 1% agarose gel to verify the correct amplicon size. Subsequently, the V3–V4 PCR amplicons were purified using the Genomic DNA Cleanup and Concentrator Kit (ZYMO RESEARCH, Irvine, CA, USA). The purified PCR products were subjected to a second round of 15 PCR cycles to add the identifiers (Nextera XT Index, Illumina, San Diego, CA, USA). The DNA concentration of the V3-V4 amplicon was determined using the Quant-it dsDNA HS Assay Kit on a Qubit fluorometer, and all samples were pooled to an equimolar concentration of 4 pM. Sequencing was performed in a paired-end modality on the Illumina MiSSeq 500 platform, generating 2 × 300 bp paired-end sequences.

### 2.5. Bioinformatic and Statistical Analysis

The paired-end raw sequences in fastq format were processed using the QIIME2pipeline (v.2023.9) [19]. Amplicon sequence variants (ASVs) were denoised and trimmed using the DADA2 plugin [20]. The taxonomic classification of ASVs was performed with the classify-consensus-v-search plugin [21] using the SILVA v138 database [22]. A phylogenetic tree of representative ASVs was constructed to calculate the UniFrac distance matrix using the plugin align-to-tree-mafft fasttree, which aligns sequences using MAFFT (v.7.520) [23] and constructs a tree using FastTree2 (v. 2.2.0) [24]. The resulting ASV frequency table and phylogeny were exported to the R environment (v.4.1.2) for further analysis. In R, the phyloseq package [25] was used to rarefy the samples to a sequencing depth of 14,000 counts. We calculated the alpha diversity indexes and Shannon and observed species, and significant differences were tested using an independent-samples *t*-test. The UniFrac distance matrix was used to calculate the beta diversity metrics and visualized in a PCoA (principal coordinate analysis). Alpha diversity was estimated using an independent-samples *t*-test. A PERMANOVA analysis was used to test for differences in beta diversity between groups using the Vegan library [26]. A linear discriminant effect size (LEfSe) analysis was performed to identify taxa with different abundances in each group, using an LDA effect cut-off of >2 and a *p*-value < 0.05. Association analyses between bacterial genera and clinical variables were performed with the MaAsLin2 package (v.3.21) [27]. Multivariable association discovery in population-scale meta-omics studies [27]. A generalized linear mixed-effects model with a negative-binomial distribution (method = ‘NEGBIN’) was specified, random intercept was the subject ID, and q < 0.01 (Benjamini–Hochberg FDR) was considered significant [27].

### 2.6. Statistical Analysis

Continuous variables were presented as the mean and standard deviation (SD), and categorical variables were presented as frequencies and percentages. For sociodemographic data, serum biochemical data, InBody analysis, lipid panel, liver panel, energy and macronutrient intake, and relative abundance of phylum, class and genera, analyses of variance (ANOVAs) and post hoc Sidak tests were performed to compare the groups (OCG, OwS, OD, OAx, and ODAx). An alpha value < 0.05 was considered significant. All statistical analyses were performed using IBM SPSS Statistics for Windows, version 21 (IBM Corp., Armonk, NY, USA).

## 3. Results

The sociodemographic characteristics of the participants are shown in Table 1. This information was obtained from the clinical assessment.

### 3.1. Body Composition, Biochemical Parameters, and Energy and Macronutrient Intake per Group

A total of 106 individuals diagnosed with obesity were included in the study. The main characteristics of the study population, stratified by the presence or absence of depressive and anxiety symptoms, are listed in Table 2. In summary, there were significant differences between the OCG and OwS groups in terms of sex, body fat %, and free body fat mass %. It is important to emphasize that the OwS group had a higher BMI compared to the control group, but without a significant difference. Significant differences were found between the stratified groups in terms of sex, BMI, muscle mass (kg), body fat %, free body fat mass %, and HDL cholesterol. Although there were no significant differences in energy intake (kcal) and the distribution of macronutrients in the diet, the OD group had a higher energy intake and consumed a higher proportion of lipids and proteins than the other groups, and OAx consumed the highest proportion of carbohydrates. Table A1 shows the significant differences between the OCG and OwS groups in terms of urea nitrogen and thyroid-stimulating hormone, and between the stratified groups in terms of body fat mass (kg), total bilirubin, indirect bilirubin, globulins, albumin and Alb/Globulin, and direct and indirect bilirubin.

### 3.2. Gut Microbiota Description

#### 3.2.1. Alpha and Beta Diversity

**Figure 1:**
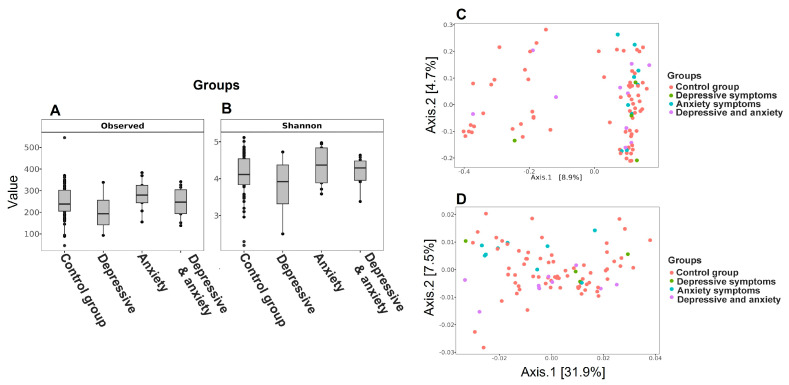
Alpha and beta diversity of the groups. (**A**) Box plot showing the alpha diversity of the bacterial communities based on the observed OTUs. (**B**) The Shannon index between the groups. (**C**) PCoA plot of the unweighted UniFrac distances and (**D**) weighted UniFrac distances of the groups using the rarefied OTU matrix. Pink dots represent the control group (OCG), green dots represent the group with depressive symptoms (OD), blue dots represent the group with anxiety symptoms (OAx), and purple dots represent the group with depressive and anxiety symptoms (ODAx).

The richness and diversity of the gut microbiota in each group are presented in Figure 1. The observed OTUs and the Shannon index (Figure 1A,B), as well as the PERMANOVA analyses of the distance matrices (Figure 1C,D), showed no significant differences in alpha and beta diversity between groups. In particular, the Shannon index showed no significant differences when comparing the OCG group with the group with depressive symptoms (OD) (*p* = 0.59), the group with anxiety symptoms (OAx) (*p* = 0.31), and the group with both symptoms (ODAx) (*p* = 0.53). The PCoA of the unweighted and weighted UniFrac distances (Figure 1C,D) also showed no clear clustering of the groups (Figure 1C,D).

#### 3.2.2. Phylum, Class, and Genus Distribution

**Figure 2:**
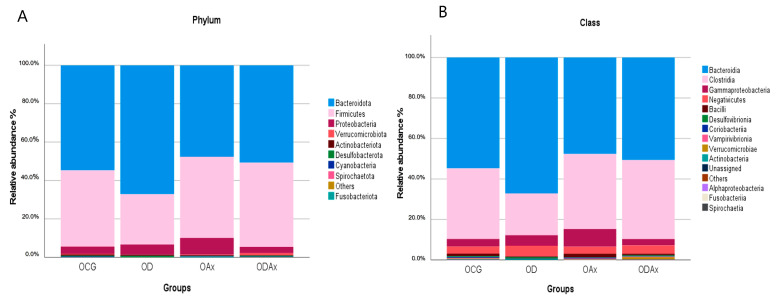
The stacked bar chart shows the relative abundances of gut bacteria at the phylum and class level. (**A**) Stacked bar chart of the classification of gut bacteria at the phylum level. (**B**) Stacked bar chart of the classification of gut bacteria at the class level. The colored bars represent different bacterial taxa identified in the bacterial profiles of the gut.

**Figure 3:**
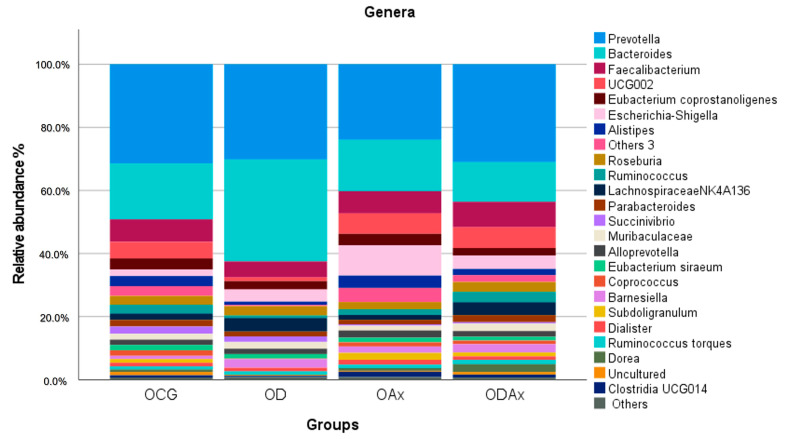
The stacked bar chart shows the relative abundances of gut bacteria at the genus level with a relative abundance >1%. Genera below these values have been included in “Others”. The colored bars represent different bacterial taxa identified in the bacterial gut profiles.

The F/B ratio showed no significant difference among groups (Table A1). The median relative abundance (MRA) at the phylum, class, and genus level are shown in Figure 2 and Figure 3. At the phylum level, the most abundant phyla in all groups were Bacteroidota (MRA = 46.9%), followed by Firmicutes (MRA = 33.32%) and Proteobacteria (MRA = 4.8%), but only Fusobacteriota—with a relative abundance of <1%—showed a significant difference among groups (*p* = 0.001) (Table A2). We found that Bacteroidota (58.82%) was the most abundant group for OD, while Firmicutes (38.59%) and Proteobacteria (8.8%) were the most abundant for OAx (Figure 2A). Notably, there were no significant differences between the groups.

At the class level, *Bacteroidia* (MRA = 47.66%) was the most common class in all groups, followed by *Clostridia* (MRA = 30.45%) and *Gammaproteobacteria* (MRA = 3.72%), although only *Gammaproteobacteria* were statistically significant (*p* = 0.042). At MRA < 1%, we found statistical differences in *Verrucomicrobiae* (*p* = 0.024) and the *Fusobacteriia* phylum (*p* < 0.001) between the groups (Table A2). Per group, we found that *Bacteroidia* (MRA = 58.82%) and *Clostridia* (MRA = 17.64%) were the predominant classes for OD, while *Clostridia* (MRA = 33.90%) and *Gammaproteobacteria* (MRA = 8.80%) were the predominant classes for OAx (Figure 2B).

In terms of genera, *Prevotella* was the most abundant genus among the groups (relative abundance = 23.03%), followed by *Bacteroides* (relative abundance = 13.45%). However, the genus with relative abundance with a significant difference was *Escherichia-Shigella* (2.32%) (*p* < 0.001), and with relative abundance <1%, it was *Clostridia* UCG014 (*p* = 0.011) (Table A2). Nevertheless, we found that *Bacteroides* (MRA = 21.22%) also dominated in OD; *Ruminococcaceae* UCG002 (MRA = 5.37%) and *Escherichia-Shigella* (MRA = 8.47%) dominated in OAx; and *Faecalibacterium* (MRA = 5.66%) dominated in ODAx (Figure 3). The post hoc analysis showed that the MRA of *Escherichia-Shigella* was significantly different between the OCG and OAx, OCG and OAx (*p* < 0.001), OD and OAx (*p* = 0.01), and OAx and ODAx groups (*p* = 0.01) (Table A2).

#### 3.2.3. Taxonomic Biomarkers in the Analyzed Groups

**Figure 4:**
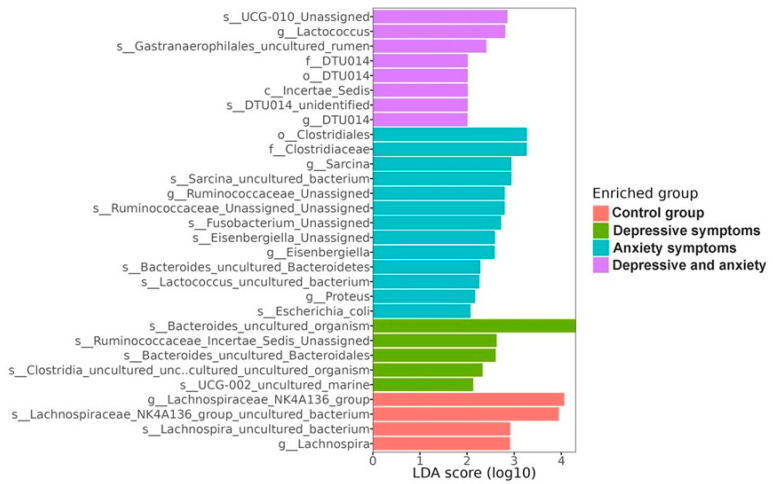
Linear discriminant effect size analysis describing the characteristic ASVs of the taxonomic profile of the control group (OCG), individuals with depressive symptoms (OD), individuals with anxiety symptoms (OAx), and individuals with both depressive and anxiety symptoms (ODAx). Only ASVs with LDA scores (log10) > 2 are shown.

Linear discriminant analysis (LDA) effect size analysis (LEfSe), which selects the strongest features between groups by combining statistical significance with relative bacterial abundance and effect relevance, showed that there are 30 taxonomic biomarkers in our population that define characteristic gut microbiota profiles between groups (Figure 4). The four OCG biomarkers were all from the Firmicutes phylum, including ASV, belonging to the genus *Lachnospiraceae* NK4A136 (*Clostridia* class and *Lachnospiraceae* family). The five biomarkers enriched in OD comprised two from the phylum Bacteroidota, including ASV, belonging to the genus *Bacteroides* (*Bacteroidia* class, *Bacteroidaceae* family); and three from the phylum Firmicutes with ASV, belonging to the genera *Ruminococcaceae* UCG-002 and *Clostridia* (both *Clostridia* class, *Ruminococcaceae* and *Clostridiaceae* family, respectively). The thirteen biomarkers that were enriched in OAx were from the phyla Firmicutes (nine ASVs), Bacteroidota (one ASV), Fusobacteroidota (one ASV), and Proteobacteria (two ASVs). It is noteworthy that this group contained ASVs that were not present in the other groups of the genera *Sarcina* (*Clostridia* class), *Eisenbergiella* (*Clostridia* class), *Lactococcus* (*Bacilli* class), *Fusobacterium* (*Fusobacteriia* class), *Proteus* (*Gammaproteobacteria* class) and *Escherichia coli* (*Enterobacteriaceae* family). The eight biomarkers that were enriched in ODAx were from the phylum *Firmicutes*, including ASVs from the genera *Ruminococcaceae* UCG-010 (*Clostridia* class, *Ruminococcaceae* family), *Lactococcus* (*Bacilli* class, *Streptococcaceae* family), and *Clostridia* DTU014 (*Clostridia* class). Of note, OD, OAx, and ODAx had *Ruminococcus* and *Clostridia* in common; OD and OAx had *Bacteroides* in common; OAx and ODAx showed an increase in *Lactococcus*; and the OAx group was uniquely enriched with *Sarcina*, *Fusobacterium*, *Eisenbergiella*, *Escherichia coli*, and *Proteus*.

#### 3.2.4. Relationship Between the Abundance of ASVs of the Taxonomic Profile of Each Studied Group and the Measured Variables

**Figure 5:**
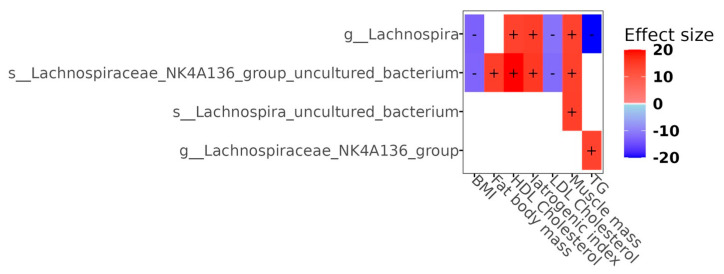
Heatmap showing significant correlations between bacterial genera, lipid panel, and body composition (*p* < 0.01). Color and +/− symbols indicate the direction and magnitude of the adjusted effects.

The associations between bacterial genus abundance, serum biochemistry, and body composition variables were analyzed using a linear mixed-effects model and the NEGBIN method (see Figure 5). The heatmap illustrates the significant associations between the bacterial genera and the body composition and lipid panel. In particular, only the genus *Lachnospira* showed positive and negative associations with these variables. The ASV belonging to the genus *Lachnospira* showed a strong positive association with HDL cholesterol, iatrogenic index, and muscle mass (kg); one ASV of the group *Lachnospiraceae* NK4A136 with body fat mass (kg), HDL cholesterol, iatrogenic index, and muscle mass; one ASV of uncultured *Lachnospira* with muscle mass; and one ASV of the genus of the group *Lachnospiraceae* NK4A136 with TG. In contrast, one ASV of the genus of *Lachnospira* was strongly negatively associated with TG.

## 4. Discussion

The relationship between obesity and depression and anxiety is bidirectional, meaning that one can influence and exacerbate the other. However, it is also complex and involves multiple factors, such as mild chronic inflammation, high levels of stress, appetite disturbances, mood swings, decreased physical activity, and the presence of a gut microbiota that is considered unhealthy [28]. Firmicutes and Bacteroidota are the most important phyla of the gut microbiota that significantly affect the host’s mental health by regulating the microbiota–gut–brain axis. Dysbiosis occurs in humans due to imbalances in the composition of the gut microbiota and leads to changes in mental health, such as the onset of anxiety and depression symptoms and other disorders [29]. Obesity has been associated with a higher proportion of *Firmicutes* and a lower proportion of Bacteroidota compared to normal-weight and lean individuals [30]; however, some studies report no significant differences in the relative abundance of Bacteroidota between obese and normal-weight people [31].

In this study, we characterized the taxonomy of the gut microbiota of adults in the Mexican Navy diagnosed with obesity with depressive and anxiety symptoms. The Mexican Navy has recognized the problem of overweight and obesity among its personnel and has implemented programs to combat this problem. These programs include weight loss programs and the examination of the gut microbiota. Navy personnel are often exposed to stressors such as irregular duty schedules, hostile environments and physical demands that can impact the gut microbiota and potentially affect mental and metabolic health [17]. Therefore, this population represents a homogeneous model of chronic stress that can help us better understand the relationship between gut health, mental health, and obesity. This could lead to new therapeutic strategies to treat these conditions in this specific population and in the general population.

We found that although the differences in the proportions of Firmicutes, Bacteroidota, and the Firmicutes/Bacteroidota ratio between the groups were not statistically significant; as expected given that our population has obesity, the relative abundance of Bacteroidota was higher and that of Firmicutes was lower. This pattern is in contrast to what has been observed in adults with obesity [30], in Mexican children with obesity [32], and in Mexican women with obesity and obesity plus metabolic disease, in whom Firmicutes were identified as the most abundant phylum, with significant changes in the abundance of the families *Ruminococcaceae*, *Lachnospiraceae*, and *Erysipelotrichaceae* [16]. However, the pattern is similar to findings in patients with depression and anxiety [28,33], although the F/B ratio was found to be higher in patients with major depressive disorder (MDD) [34]. In this sense, in a cohort of patients with depressive symptoms in a hospital in Zhejiang, China, increased bacterial alpha diversity was found in the stool compared to healthy controls. At the phylum level, the relative abundance of Bacteroidota, Proteobacteria, and Actinobacteria was increased, while Firmicutes was significantly reduced. Despite considerable interindividual variability, several predominant bacterial genera differed significantly between individuals with depressive symptoms and controls. In particular, patients with depressive symptoms showed increased levels of *Enterobacteriaceae* and *Alistipes*, in addition to a significant reduction in *Faecalibacterium* [35]. In a separate cohort from the same hospital, patients diagnosed with GAD had significantly lower microbial diversity compared to healthy controls, as measured by observed operational taxonomic units (OTUs) and Simpson, ACE and Chao indices. The relative abundances of nine bacterial genera differed significantly between the two groups. *Faecalibacterium*, *Eubacterium rectale*, *Roseburia*, *Subdoligranulum*, and *Lachnospira* were more abundant in the control group, while *Bacteroides*, *Escherichia*-*Shigella*, *Ruminococcus gnavus*, *Lactobacillus*, and *Fusobacterium* were enriched in patients with anxiety. Of note, the GAD group showed bacterial overgrowth with potentially pathogenic genera, including *Escherichia*-*Shigella*, *Fusobacterium*, and *Ruminococcus gnavus*, compared to healthy individuals [36]. In a study conducted among Chinese people living in Beijing, patients diagnosed with MDD had significantly lower alpha diversity indices compared to healthy controls. The Shannon index indicated that microbial richness was significantly higher in the control group, while Faith’s phylogenetic diversity index indicated that phylogenetic diversity was also significantly higher in the control group. At the phylum level, Firmicutes was most reduced in patients with MDD. The study identified 13 taxonomic biomarkers within the Firmicutes phylum. In the control group, enriched taxa included members of the *Lachnospiraceae* and *Ruminococcaceae* families, as well as the genera *Coprococcus*, *Blautia*, *Clostridiaceae*, and *Dorea*. In contrast, the MDD group showed an enrichment of taxa from the phyla Proteobacteria (e.g., *Oxalobacter* and *Pseudomonas*) and Firmicutes (e.g., *Parvimonas*, *Bulleidia*, *Peptostreptococcus*, and *Gemella*). The authors concluded that Firmicutes is the most important phylum associated with depressive symptoms [33]. Patients diagnosed with GAD in a hospital in Xi’an, China, had a lower number of operational taxonomic units (OTUs) and significantly lower bacterial alpha diversity in their stools compared to healthy controls. At the phylum level, the relative abundance of Firmicutes and Tenericutes was significantly lower in the GAD group. In addition, several bacterial genera were differentially represented in the patients and control subjects [37]. The discrepancies between these results may be due to factors such as small sample sizes, different inclusion criteria, disease duration, and severity, but the fact is that an imbalance in the gut microbiota contributes to the development of depression. The lower proportion of Firmicutes could contribute to a decrease in SCFA-producing bacteria, which may play an important role in the low-grade inflammation observed in depression [33]. In addition, the higher proportion of Bacteroidota and *Fusobacteriia*, together with the excessive growth of *Escherichia-Shigella*, *Fusobacterium*, and *Ruminococcus gnavus*, could contribute to the symptomatology of anxiety [36], as well as factors such as diet, stress, genetics, and medication use (especially antibiotics) that also alter the composition of the gut microbiota, which in turn can influence the onset and severity of these disorders [6]

In this study, we also reported that the enterotype of Mexican adults with obesity with or without symptoms of depression and/or anxiety was *Prevotella*, as previously reported for some populations [37,38]. This enterotype is characterized by its ability to produce more SCFAs due to its greater fermentation capacity [39]; therefore, it is considered beneficial for gut health and is often associated with a high-fiber, high-carbohydrate diet, which is prevalent in the traditional Mexican diet [18,40]. However, its excessive growth may contribute to the occurrence of depression and anxiety symptoms, as it has pro-inflammatory effects, and the deregulation of its growth is associated with the development of dysbiosis [41] and obesity [42]. In contrast, some studies have reported that the presence of *Prevotella* in combination with *Eubacterium coprostanoligenes* is negatively correlated with the severity of anxiety symptoms and positively correlated with a reduction in these symptoms [37]. In addition, other studies suggest that higher levels of *Prevotella* are associated with lower levels of anxiety and depression [36]. In Mexico, a higher abundance of *Prevotella* was only found in individuals with metabolic syndrome [43], with the salivary amylase gene (AMY1)—an enzyme that can influence the composition of the gut microbiota by breaking down carbohydrates, especially starch [44]—in obese adults and in Mexican people with type 2 diabetes [15]. Our results show that *Prevotella* is not associated with either depressive or anxiety symptoms in Mexican adults with obesity, as neither the depressed nor the anxious groups were enriched with this genus.

Our LEfSe analysis, in which we compared the relative abundances of bacteria from the OCG, OD, OAx and ODAx groups, showed that each group was enriched with bacterial genera that define 30 taxonomic biomarkers that are critical for defining gut microbiota profiles that allow differentiation between obese individuals with depressive and anxiety symptoms and those without these symptoms. One of the key findings of this work was that the OCG group showed an exclusive increase in the genus *Lachnospira* (*Lachnospira* uncultured group and *Lachnospira* NK4A136), making it a potential biomarker for obesity, not associated with depressive or anxiety symptoms, although interestingly, one study reported that the genus *Lachnospiraceae* NK4A136 is an ASV biomarker for leanness status in humans in Spain [45]. In this sense, the absence of depressive and anxious symptoms in our OCG can be related to the production of butyrate and acetate by *Lachnospira*. Both SCFAs can be absorbed by the host and contribute to higher caloric intake from food, leading to weight gain and obesity [46], while also strengthening the intestinal barrier and contributing to proper colonocyte function, reducing intestinal permeability and preventing lipopolysaccharide (LPS) from entering the bloodstream, thereby preventing systemic inflammatory processes and possibly promoting an increase in brain-derived neurotrophic factor (BDNF), a crucial factor involved in brain plasticity, mood regulation, and cognitive function [47]. Thus, in vivo and in vitro studies have shown that butyrate can counteract cognitive decline and loss of neuronal spines and prevent low BDNF levels by maintaining gut integrity [48]. Low BDNF levels are associated with depression and anxiety, so the ability of butyrate-producing bacteria to increase BDNF expression may be one mechanism by which they exert and maintain antidepressant and anxiolytic effects in the OCG [28,29,38,40,47,48,49,50]. Therefore, the determination of butyrate and BDNF levels could confirm their involvement in brain plasticity, mood regulation, and cognitive functions [29,40,45,46,47,48,49,50]. The OD, OAx, and ODAx groups were enriched with genera of *Ruminococcus* (UCG 010, unassigned *Ruminococcus*, and *Ruminococcus Incertae sedis*) and two species of the genus *Clostridia* (uncultured *Clostridia* and unclassified DTU014). *Ruminococcus* and *Clostridia* are butyrate-producing bacteria that play a crucial role in cognitive function and in the regulation of anxiety, depression, and stress-related behavior by maintaining gut integrity and reducing inflammation [36,51,52,53,54]. The OD and ODAx groups only shared the genus *Bacteroides*, which elicits various effects that modulate the occurrence of depression-like behavior [55] and anxiety [56]. *Bacteroides* play a crucial role in the production of acetate and propionate [57], SCFAs that have anti-inflammatory effects, maintain gut barrier integrity, modulate the gut–brain axis, and influence mood by reducing inflammation and supporting healthy brain function [58,59]. However, *Bacteroides* induce several effects that modulate the occurrence of depression-like behaviors. These effects are initially attributed in part to the influence of these bacterial populations on the production of metabolites of the tryptophan pathway and some neurotransmitters along the gut–brain axis. This has been shown to lead to a particularly significant reduction in serotonin levels in the brain, which is associated with an increased susceptibility to the onset of depressive moods. In this context, only a few selected species of *Bacteroides* (*B. thetaiotaomicron*, *B. fragilis*, and *B. uniformis*) have been shown to contribute to this susceptibility to depression in mouse models [55]. Secondly, to their ability to inhibit the growth of Firmicutes [60], and thirdly, to their positive association with the severity of anxiety [37,55,60]. The OAx and ODAx groups showed an exclusive increase in the genus *Lactococcus*. This genus has been mainly associated with an anti-obesity effect [61], but some authors suggest that it plays an important role in obesity, as it contributes significantly to obesity, mainly through the production of butyrate, which, as mentioned above, provides additional energy to the host and causes low-grade inflammation [62,63,64,65]. In addition, certain species, such as *L. lactis*, influence the production of neurotransmitters such as gamma-aminobutyric acid (GABA), an important inhibitory neurotransmitter in the central nervous system, and the modulation of inflammatory responses, both of which are important key factors in anxiety [62].

The OAx group was exclusively enriched with the genera *Sarcina*, *Fusobacterium*, *Eisengergiella*, *Escherichia coli*, and *Proteus*. The role of *Sarcina* in depression and anxiety is not well documented, but its presence in the gastrointestinal tract is associated with abdominal symptoms (pain, nausea, vomiting, diarrhea, and dyspepsia) with delayed gastric emptying or gastric obstruction [66]. *Fusobacterium* is considered a pathogen due to its invasive and inflammatory properties, its ability to invade the bloodstream and disrupt the intestinal barrier, and its involvement in triggering immune responses associated with generalized anxiety disorder (GAD) and functional gastrointestinal disorders. GAD may be associated with an increase in the relative abundance of *Fusobacterium* [67], and although *Eisenbergiella* is considered part of the normal gut flora, changes in its abundance or activity may be associated with dysbiosis, which can lead to various health problems, including gastrointestinal disease and colorectal cancer [68], and it contributes to the pathophysiology of anxiety and depression by disrupting gut–brain communication. The significant prevalence of the *Escherichia-Shigella* group in our population, especially in individuals with anxiety symptoms, is consistent with previous reports [37,69]. *Escherichia coli* and *Shigella* species are associated with symptoms of gastrointestinal infections and may be markers of dysbiosis. However, recent studies have investigated the possible involvement of these bacteria in depression and anxiety, as their presence is associated with the production of pro-inflammatory cytokines in the gut that affect brain function and mood. In addition, these bacteria produce high levels of LPS, which can increase the permeability of the blood–brain barrier, increase neuroinflammation, cross the intestinal barrier, and enter the bloodstream during dysbiosis, leading to systemic inflammation. Patients with GAD have a significantly higher proportion of *Escherichia-Shigella* compared to patients without GAD [69,70]. The pathogenesis of GAD is not yet fully understood, but a dysregulation of neurotransmitters, particularly serotonin and GABA, is suspected [37]. Certain bacteria, such as *Escherichia*, can produce serotonin, GABA, dopamine, and norepinephrine, indicating a possible role of the gut microbiota in the modulation of anxiety [54]. Research suggests that fecal microbiota transplantation (FMT) can significantly reduce *Escherichia-Shigella* levels in patients, potentially impacting anxiety via serotonergic signaling pathways [69]. However, further studies are needed to investigate the effects of FMT on the composition of the gut microbiome and its therapeutic potential in GAD.

Given that metabolic dysregulation may contribute to the development of depression and anxiety [4], the altered microbial compositions observed in our population with obesity were only associated with the dysregulation of fat metabolism and body composition. We determined the significant associations between the abundance of ASVs of the taxonomic profile of each studied group with the lipid panel and body composition. The results reveal complex interactions between the gut microbiota and physiological factors and emphasize the potential role of the microbiome in metabolic health, as only the genus *Lachnospira* showed a positive and negative association with these variables. The strong positive association of *Lachnospiraceae* NK4A136 with muscle mass suggests that these bacteria may contribute to the maintenance of lean body mass, as these genera can influence muscle metabolism through the production of butyrate, which is known to play a role in muscle function and overall metabolic health [45,71]. Furthermore, the positive association of *Lachnospiraceae* NK4A136 with HDL cholesterol and the iatrogenic index underscores its versatile role in both lipid metabolism and response to medical interventions [45]. Furthermore, the negative association of the genus *Lachnospira* with TG emphasizes the role of these bacteria in lipid metabolism. This relationship may contribute to lipid homeostasis and potentially influence cardiovascular risk factors and energy expenditure. In addition, *Lachnospira* may modulate metabolic pathways that counteract weight gain [45].

One of the main limitations of this work is that we did not have a lean control group without symptoms of depression and anxiety to determine whether the observed biomarkers of the gut microbiota of obese individuals are specific to each stratified group. The second limitation is that we did not have access to cutoff points to determine the severity of depressive and anxiety symptoms to define whether the taxonomic biomarkers might be influenced by symptom severity in all participants. The third limitation is that as women are more susceptible to depression and anxiety, it would be very interesting for future research in our study population to determine how hormonal fluctuations, the menstrual cycle, pregnancy, the postpartum period, and menopause might influence the gut microbiota, and thus modulate susceptibility to mood disorders. The fourth limitation of the study is the unequal sample size of the different groups; however, it is important to emphasize that the participants who were included in this study were those who were willing to participate in the study, regardless of whether or not they exhibited any mood symptomatology. Furthermore, the groups were only statistically significantly heterogeneous in terms of the proportion of men and women. As the population studied was naval personnel, the lower presence of women is to be expected and is not considered a bias in the study. Considering that 16S rRNA sequencing only provides a taxonomic snapshot and does not directly capture functional or metabolic activity, it will be necessary to incorporate shotgun metagenomics and untargeted metabolomics in a future work to elucidate the mechanistic links between the gut microbiota and host mental health.

## 5. Conclusions

Our study is the first to identify distinct gut microbiota profiles in Mexican adults with obesity and depressive and anxiety symptoms. Each group showed enrichment in specific bacterial genera, resulting in 30 taxonomic biomarkers that distinguish between obese individuals with and without these symptoms. This distinction offers potential clinical utility for the diagnosis and treatment of depression and anxiety in obese populations.

## Figures and Tables

**Table 1:** Sociodemographic characteristics of the population.

Variables	Total
Age	
Years	39.44
Mean (±SD)	(±7.32)
Sex	
Male (%)	83 (78.3)
Female (%)	23 (21.7)
Schooling	
- Middle school (%)	30 (28.6)
- High school (%)	25 (23.8)
- Professional technician (%)	15 (14.3)
- Bachelor’s degree (%)	25 (23.8)
- Master’s degree (%)	6 (5.7)
- Specialty (%)	2 (1.9)
Marital Status	
- Married (%)	68 (64.8)
- Single (%)	22 (21)
- Unmarried (%)	11 (10.5)
- Divorced (%)	4 (3.8)

**Table 2:** Serum biochemistry, body composition, energy intake, and diet macronutrient distribution.

Stratified Groups								
Variables	OCG (n = 79)	OwS (n = 27)	*p*	OD (n = 7)	OAx (n = 8)	ODAx (n = 12)	Total (n = 106)	*p*
Sex								
Male (%)	67 (84.8)	15 (60)	**0.01**	3 (50%)	7 (70)	6 (54.5)	83 (78.3)	**0.03**
Female (%)	12 (15.2)	10 (40)		3 (50%)	3 (30)	5 (45.5)	23 (21.7)	
Age (years)	39.96	37.41	0.20	41.83	36.80	36.82	39.44	0.29
Mean (±SD)	(7.61)	(6.15)		(7.94)	(6.18)	(3.73)	(7.32)	
**Serum biochemistry**								
Glucose (mg/dL)	95.02	89.71	0.26	94.20	87.72	90.18	93.8	0.71
Mean (±SD)	(21.94)	(14.45)		(15.80)	(17.05)	(10.82)	(20.51)	
HDL cholesterol (mg/dL)	41.05	40.80	0.91	46.67	40.50	40.45	40.99	0.57
Mean (±SD)	(10.15)	(9.89)		(12.17)	(10.55)	(11.74)	(10.05)	
LDL cholesterol (mg/dL)	128.45	132.36	0.62	142.67	138.90	118.27	129.38	0.43
Mean (±SD)	(34.70)	(35.41)		(52.12)	(21.95)	(33.25)	(34.74)	
Total cholesterol (mg/dL)	207.45	203.88	0.70	220.17	206.70	192.91	206.60	0.59
Mean (±SD)	(40.87)	(41.94)		(59.48)	(31.66)	(36.66)	(40.95)	
TG (mg/dL)	175.84	155.80	0.30	154.67	141.70	171	171.07	0.63
Mean (±SD)	(87.80)	(70.54)		(50.66)	(74.18)	(75.18)	(84.131)	
**Body composition analysis**								
Weight (Kg)	97.02	96.85	0.94	100.46	98.78	93.35	96.98	0.56
Mean (±SD)	(10.91)	(11.37)		(10.39)	(10.51)	(12.09)	(10.96)	
BMI (kg/m^2^)	34.53	35.82	0.07	39.65	34.35	34.67	34.84	**0.002**
Mean (±SD)	(3.06)	(3.52)		(2.69) ^a,b,c^	(2.38)	(0.98)	(3.2)	
Body fat (%)	37.41	40.45	**0.03**	43.89	39.15	40.18	38.13	**0.04**
Mean (±SD)	(5.91)	(6.80)		(6.04)	(6.68)	(7.24)	(6.23)	
Free body fat mass %	62.59	59.55	**0.03**	56.10	60.85	59.82	61.87	**0.04**
Mean (±SD)	(5.91)	(6.80)		(6.04)	(6.68)	(7.24)	(6.24)	
**Energy and macronutrients intake**								
Energy (kcal/day)	2711.68	2780.56	0.76	2947.67	2607	2704	2727.92	0.93
Mean (±SD)	(1009.45)	(971.70)		(970.65)	(892.207)	(1099.42)	(996.52)	
Protein percentage	18.77	18.96	0.89	21.83	17.80	19	18.82	0.63
Mean (±SD)	(5.76)	(7.44)		(13.87)	(2.61)	(5.11)	(6.1)	
Lipid percentage	30.34	30.83	0.81	31.80	28.40	33.64	30.46	0.56
Mean (±SD)	(9.09)	(8.67)		(7.05)	(9.75)	(9.77)	(8.96)	
Carbohydrate percentage	50.76	50	0.77	46.67	53.70	46.82	50.58	0.43
Mean (±SD)	(11.42)	(11.58)		(13.21)	(10.39)	(12.28)	(11.41)	

Obese control group, OCG; obese with depressive and/or anxiety symptoms, OwS; obese with depressive symptoms, OD; obese with anxiety symptoms, OAx; obese with depressive and anxiety symptoms, ODAx. Text in bold denotes statistical significance. ^a^: Significant *p*-value obtained from post hoc test of OD against OCG. ^b^: Significant *p*-value obtained from post hoc test of OD against OAx. ^c^: Significant *p*-value obtained from post hoc test of OD against ODAx.

**Figure A1:**
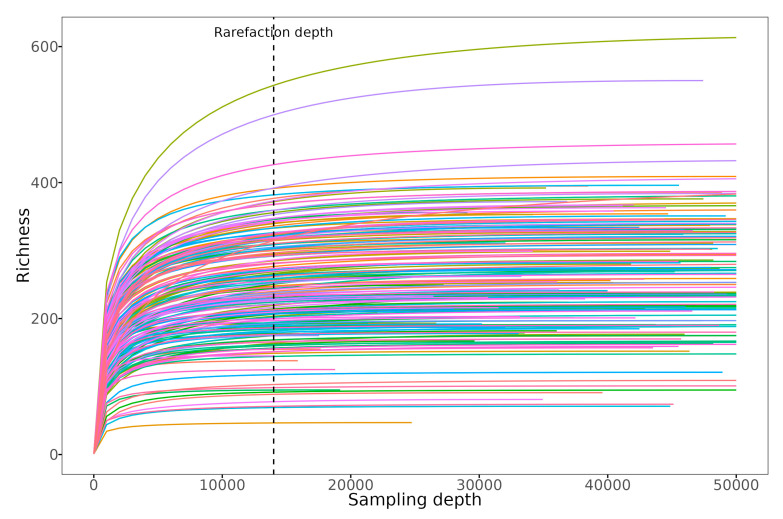
Rarefaction curves.

## Data Availability

The datasets generated and/or analyzed are available from the corresponding author. The raw data has been submitted to the NCBI platform and is publicly available at BioProject ID: PRJNA1289340. The original data presented in the study are openly available in the NCBI’s Sequence Read Archive (SRA) at [http://www.ncbi.nlm.nih.gov/bioproject/1289340] or [accession number PRJNA1289340] (accessed on 9 July 2025).

## Appendix A

**Table A1:** Supplementary results of serum biochemistry and body composition of the studied population.

Stratified Groups								
Variables	OCG (n = 79)	OwS (n = 25)	*p*	OD (n = 7)	OAx (n = 8)	ODAx (n = 12)	Total (n = 106)	*p*
**Serum Biochemistry**								
Ureic nitrogen (mg/dL)	15.71	27.22	**0.03**	23.64	28.06	25.79	18.39	0.27
Mean (±SD)	(19.35)	(32.18)		(28.89)	(33.32)	(33.33)	(23.32)	
Creatinine (mg/dL)	0.90	0.87	0.38	0.91	0.86	0.84	0.89	0.55
Mean (±SD)	(0.16)	(0.17)		(0.17)	(0.15)	(0.18)	(0.161)	
C-reactive protein (mg/dL)	0.52	0.83	0.09	0.55	0.68	1.10	0.60	0.12
Mean (±SD)	(0.63)	(1.11)		(0.57)	(0.52)	(1.57)	(0.77)	
Uric acid (mg/dL)	6.75	6.35	0.28	5.77	6.51	6.51	6.65	0.51
Mean (±SD)	(1.57)	(1.78)		(1.33)	(1.56)	(2.41)	(1.62)	
Insulin (Uu/mL)	11.74	13.11	0.27	12.84	14.53	11.96	12.08	0.48
Mean (±SD)	(5.69)	(4.50)		(5.67)	(4.67)	(3.07)	(5.44)	
Cortisol (µg/dL)	8.64	8.88	0.75	8.69	8.87	8.59	8.7	0.99
Mean (±SD)	(3.45)	(2.71)		(3.4)	(2.44)	(2.73)	(3.28)	
Thyroid-stimulating hormone	2.58	4.26	**0.01**	4.50	2.64	4.50	2.91	0.07
(TSH) (uUI/mL)	(1.63)	(3.58)		(2.10)	(1.57)	(5)	(2.22)	
Iatrogenic index	5.24	5.12	0.71	4.83	5.20	5.09	5.21	0.89
Mean (±SD)	(1.37)	(1.36)		(1.72)	(1.23)	(1.45)	(1.36)	
**InBody analysis**								
Body fat mass (Kg)	36.43	39.73	0.07	46.31	38.63	37.45	37.21	**0.02**
Mean (±SD)	(7.67)	(8.64)		(7.44)	(7.31)	(8.51)	(7.99)	
Free body fat mass (Kg)	60.63	57.12	0.06	54.15	60.14	55.89	59.80	0.10
Mean (±SD)	(7.89)	(9.24)		(7.95)	(9.29)	(10.14)	(8.32)	
Muscle mass (Kg)	32.33	30.80	0.21	32.50	30.90	28.91	31.97	0.19
Mean (±SD)	(5.21)	(5.91)		(5.78)	(6.23)	(5.59)	(5.39)	
**Liver panel**								
Total bilirubin (mg/dL)	1.11	0.78	**<0.001**	0.78	0.85	0.83	1.04	**0.014**
Mean (±SD)	(0.39)	(0.21)		(0.17)	(0.25)	(0.34)	(0.39)	
Direct bilirubin (mg/dL)	0.130	0.104	0.212	0.08	0.12	0.09	0.124	0.33
Mean (±SD)	(0.09)	(.06)		(0.04)	(0.09)	(0.03)	(0.08)	
Indirect bilirubin (mg/dL)	0.985	0.683	**<0.001**	0.70	0.73	0.74	0.91	**0.011**
Mean (±SD)	(0.34)	(0.18)		(0.14)	(0.18)	(0.35)	(0.34)	
AST (UI/L)	37.59	30.88	0.31	22.33	36.78	30.55	36.04	0.55
Mean (±SD)	(30.68)	(17.59)		(5.20)	(24.36)	(13.92)	(28.27)	
ALT (UI/L)	58.65	42.79	0.20	22	61.44	40.91	54.99	0.31
Mean (±SD)	(55.97)	(41.87)		(6.22)	(61.55)	(28.29)	(53.28)	
Total proteins (g/dL)	7.31	7.26	0.58	7.17	7.36	7.25	7.30	0.80
Mean (±SD)	(0.34)	(0.53)		(0.74)	(0.40)	(0.49)	(0.39)	
Albumin (g/dL)	4.33	4.16	**0.006**	3.95	4.23	4.21	4.29	**0.003**
Mean (±SD)	(0.23)	(0.32)		(0.41) ^a^	(0.25)	(0.27)	(0.27)	
Globulins (mg/dL)	3	3.13	0.13	3.22	3.12	3.11	3.03	0.30
Mean (±SD)	(0.36)	(0.334)		(0.37)	(0.32)	(0.32)	(0.35)	
Alb/Globulin	1.47	1.35	**0.007**	1.23	1.37	1.37	1.44	**0.008**
Mean (±SD)	(0.21)	(0.17)		(0.09) ^a^	(0.166)	(0.19)	(0.21)	
GGT (UI/L)	45.66	38.25	0.40	27	44.78	36.45	43.95	0.60
Mean (±SD)	(41.27)	(24.29)		(18.34)	(16.47)	(30.12)	(18.05)	
AP (UI/L)	64.40	68.46	0.57	73.67	68.56	66.36	66.88	0.70
Mean (±SD)	(16.45)	(11.61)		(12.09)	(13.19)	(9.5)	(15.44)	

Aspartate aminotransferase, AST; alanine aminotransferase, ALT; gamma glutamyl transferase, GGT; alkaline phosphatase, AP; obese control group, OCG; obese with depressive and/or anxiety symptoms group, OwS; obese with depressive symptoms group, OD; obese with anxiety symptoms group, OAx; obese with depressive and anxiety symptoms group, ODAx. Text in bold denotes statistical significance. ^a^: Significant *p*-value obtained from post hoc test of OD against OCG.

## Appendix B

**Table A2:** Distribution of phylum, class, and genera of the gut microbiota among groups.

Variable	OCG (n = 79)	OD (n = 7)	OAx (n = 8)	ODAx (n = 12)	Total (n = 106)	*p*
**Phylum**						
F/B	0.79 (0.71)	0.44 (0.62)	1.0 (0.80)	0.79 (0.71)	0.79 (0.72)	0.48
Bacteroidota	48.50 (23.10)	58.82 (31.81)	38.84 (20.11)	41.46 (22.55)	47.66 (23.53)	0.30
Firmicutes	35.15 (19.81)	21.81 (18.75)	38.59 (21.61)	37.75 (20.09)	34.75 (19.95)	0.33
Proteobacteria	3.39 (6.76) ^b^	4.03 (4.33)	8.80 (14.61)	3.00 (3.54)	3.8 (7.26)	0.24
Verrucomicrobiota	0.25 (0.82) ^c^	0.03 (0.040) ^d^	0.12 (0.165)	0.83 (1.35)	0.29 (0.85)	0.11
Actinobacteriota	0.63 (1.22)	0.56 (1.00)	0.49 (0.49)	0.29 (0.24)	0.58 (1.10)	0.83
Desulfobacterota	0.43 (0.53)	0.51 (0.71)	0.18 (0.23)	0.47 (0.58)	0.42 (0.53)	0.66
Cyanobacteria	0.27 (0.92)	0.008 (0.014)	0.28 (0.79)	0.18 (0.36)	0.24 (0.83)	0.86
Spirochaetota	0.003 (0.014)	0.000 (0.000)	0.001 (0.005)	0.009 (0.025)	0.003 (0.01)	0.58
Otras	0.05(0.089)	0.009 (0.024)	0.05 (0.091)	0.07 (0.18)	0.04 (0.099)	0.60
Fusobacteriota	0.01 (0.05) ^b^	0.078 (0.14)	0.18 (0.36) ^e^	0.003 (0.008)	0.03 (0.11)	**0.001**
**Class**						
*Bacteroidia*	48.50 (23.10)	58.82 (31.81)	38.84 (20.11)	41.46 (22.55)	47.66 (23.53)	0.30
*Clostridia*	30.90 (18.62)	17.64 (15.60)	33.90 (19.92)	32.73 (18.70)	30.45 (18.63)	0.29
*Gammaproteobacteria*	3.29 (6.77) ^b^	4.03 (4.33)	8.80 (14.61)	2.93 (3.50)	3.72 (7.27)	**0.04**
*Negativicutes*	3.10 (3.42)	3.76 (4.30)	3.30 (2.7)	3.33 (4.02)	3.19 (3.46)	0.97
*Bacilli*	1.12 (1.17)	0.39 (0.47)	1.37 (1.12)	1.00 (1.39)	1.08 (1.16)	0.38
*Desulfovibrionia*	0.38 (0.52)	0.43 (0.67)	0.16 (0.22)	0.39 (0.55)	0.37 (0.51)	0.7
*Coriobacteriia*	0.34 (0.65)	0.19 (0.31)	0.34 (0.43)	0.19 (0.21)	0.32 (0.58)	0.78
*Vampirivibrionia*	0.27 (0.92)	0.008 (0.014)	0.28 (0.79)	0.18 (0.36)	0.24 (0.83)	0.863
*Verrucomicrobiae*	0.20 (0.80) ^c^	0.02 (0.04) ^e^	0.04 (0.06)	0.79 (1.32)	0.25 (0.84)	**0.02**
*Actinobacteria*	0.21 (0.57)	0.28 (0.63)	0.089 (0.079)	0.054 (0.072)	0.19 (0.51)	0.67
*No asignado*	0.15 (0.05)	0	0.003 (0.010)	0.02 (0.07)	0.01 (0.05)	0.71
*Otros*	0.10 (0.14)	0.01 (0.02)	0.13 (0.17)	0.12 (0.20)	0.010 (0.14)	0.37
*Alphaproteobacteria*	0.096 (0.33)	0.001 (0.002)	0	0.069 (0.21)	0.07 (0.29)	0.72
*Fusobacteriia*	0.019 (0.054) ^b^	0.07 (0.14)	0.18 (0.36)	0.003 (0.008) ^e^	0.03 (0.11)	**<0.001**
*Spirochaetia*	0.003 (0.14)	0	0.001 (0.005)	0.008 (0.024)	0.003 (0.014)	0.64
**Genera**						
*Prevotella*	23.82 (22.14)	26.83 (33.00)	14.16 (14.37)	21.49 (20.16)	23.03 (22.15)	0.65
*Bacteroides*	13.39 (16.15)	21.22 (24.14)	13.81 (11.52)	9.06 (8.54)	13.45 (15.8)	0.46
*Faecalibaterium*	5.44 (5.20)	4.16 (5.17)	5.36 (3.98)	5.66 (4.26)	5.38 (4.97)	0.92
*UCG 002*	3.94 (4.07) ^a^	0.87 (0.92)	5.37 (3.65)	4.63 (3.87) ^d,f^	3.92 (3.96)	0.13
*Eubacterium coprostalinogenes*	2.69 (2.93)	1.93 (1.80)	2.95 (2.47)	1.83 (2.43)	2.57 (2.78)	0.68
*Alistipes*	2.42 (3.47)	0.75 (0.86)	3.37 (3.74)	1.49 (2.35)	2.28 (3.29)	0.37
*Otros*	2.35 (3.51)	2.04 (4.66)	2.32 (1.88)	1.69 (1.68)	2.25 (3.31)	0.93
*Roseburia*	2.14 (2.78)	2.03 (4.56)	1.85 (1.79)	2.18 (2.10)	2.12 (2.76)	0.99
*Ruminococcus*	2.07 (2.04)	0.66 (0.58)	1.28 (1.19)	2.28 (2.26)	1.94 (1.97)	0.21
*Succinivibrio*	1.73 (6.03)	1.18 (0.96)	0.35 (0.96)	0.28 (0.52)	1.42 (5.24)	0.76
*Escherichia-Shigella*	1.55 (2.30) ^b^	2.54 (2.90)	8.47 (14.20)	3.18 (3.81) ^e,f^	2.32 (4.78)	**<0.001**
*Lachnospiraceae NK4A136*	1.52 (2.03)	2.87 (5.79)	1.28 (0.84)	2.64 (3.93)	1.72 (2.62)	0.32
*Parabacteroides*	1.60 (1.55)	1.21 (0.80)	1.06 (0.63)	1.51 (1.38)	1.52 (1.44)	0.73
*Muribaculaceae*	1.40 (2.79)	1.46 (3.59)	1.36 (2.35)	1.52 (2.53)	1.41 (2.75)	0.99
*Alloprevotella*	1.30 (1.89)	1.09 (1.73)	2.15 (3.25)	1.18 (1.90)	1.34 (1.99)	0.68
*Coprococcus*	1.30 (1.48) ^a^	0.22 (0.33)	0.91 (1.02)	0.87 (0.95)	1.15 (1.37)	0.19
*Eubacterium siraeum*	1.26 (1.86)	0.95 (1.23)	1.04 (1.45)	1.03 (1.09)	1.20 (1.71)	0.93
*Dialister*	1.00 (2.76)	0.86 (1.67)	1.67 (2.23)	0.89 (2.05)	1.03 (2.57)	0.92
*Subdoligranulum*	0.90 (1.65)	1.00 (2.59)	1.17 (1.08)	0.84 (1.08)	0.92 (1.61)	0.97
*Uncultured*	0.88 (1.73)	0.25 (0.44)	0.25 (0.36)	0.63 (1.55)	0.76 (1.55)	0.60
*Ruminococcus torques*	0.81 (1.44)	0.80 (0.64)	1.06 (0.56)	1.11 (1.83)	0.86 (1.39)	0.90
*Barnesiella*	0.73 (1.81)	1.74 (3.21)	1.86 (3.99)	1.66 (5.42)	0.99 (2.71)	0.43
*Dorea*	0.69 (1.56) ^c^	0.06 (0.14)	0.75 (0.65)	2.02 (4.11) ^d^	0.80 (1.93)	0.16
*Clostridia UCG014*	0.61 (0.67) ^b,f^	0.45 (0.35)	1.71(1.82)	0.73 (1.07) ^e^	0.7 (0.87)	**0.01**
*UCG003*	0.64 (0.65)	0.95 (1.20)	0.62 (0.30)	0.68 (0.43)	0.66 (0.65)	0.75
*Rikenellaceae RC9gut group*	0.73 (1.80)	0.20 (0.28)	0.10 (0.15)	0.42 (0.65)	0.62 (1.59)	0.65
*UCG005*	0.61 (0.61)	0.19 (0.22)	0.54 (0.35)	0.64 (0.47)	0.58 (0.56)	0.37
*NK4A214 group*	0.54 (0.64)	0.46 (0.54)	0.78 (0.50)	0.52 (0.44)	0.55 (0.60)	0.77
*Paraprevotella*	0.53 (0.70)	0.42 (0.59)	0.46 (0.41)	0.85 (1.89)	0.55 (0.79)	0.63
*Phascolarctobacterium*	0.49 (0.56)	0.26 (0.19)	0.73 (0.88)	0.97 (1.66)	0.54 (0.77)	0.2
*Uncultured X*	0.55 (0.46) ^a^	0.033 (0.048) ^a^	0.59 (0.51) ^f^	0.56 (0.30) ^d^	0.52 (0.45)	**0.04**
*Blautia*	0.49 (0.97)	0.003 (0.005)	0.54 (0.53)	0.66 (0.72)	0.48 (0.89)	0.59
*Veillonella*	0.51 (1.18)	0.13 (0.28)	0.15 (0.17)	0.35 (0.55)	0.44 (1.05)	0.70
*Christensenellaceae R7 group*	0.40 (0.55)	0.013(0.032)	0.50 (0.70)	0.80 (1.15) ^d^	0.42 (0.64)	0.11
*Uncultured A*	0.37 (0.74) ^c^	0.03 (0.071)	0.38 (0.25)	0.92 (1.15) ^d^	0.41 (0.76)	0.10
*Akkermansia*	0.37 (0.87) ^c^	0.09 (0.17)	0.06 (0.12)	0.98 (1.39) ^e^	0.40 (0.90)	0.11
*UCG010*	0.39 (0.42)	0.16 (0.27)	0.55 (0.34)	0.44 (0.33)	0.39 (0.40)	0.37
*Gastranaerophilales*	0.41 (1.56)	0	0.32 (0.84)	0.23 (0.41)	0.35 (1.37)	0.89
*Haemophilus*	0.40 (0.83)	0.17 (0.15)	0.22 (0.33)	0.09 (0.093)	0.34 (0.73)	0.53
*Streptococcus*	0.35 (0.45)	0.147 (0.146)	0.20 (0.23)	0.39 (0.83)	0.33 (0.47)	0.65
*Unassigned*	0.33 (0.33)	0.15 (0.094)	0.41 (0.35)	0.26 (0.23)	0.32 (0.31)	0.42
*Catenibacterium*	0.30 (0.38) ^b^	0.053 (0.064) ^f^	0.77 (0.78) ^b^	0.25 (0.32) ^e^	0.31 (0.42)	**0.01**
*Desulfovibrio*	0.33 (0.50)	0.14 (0.22)	0.10 (0.17)	0.30 (0.33)	0.30 (0.45)	0.48
*Butyrivibrio*	0.29 (0.70)	0.26 (0.42)	0.31 (0.56)	0.38 (1.14)	0.30 (0.73)	0.98
*Sutterella*	0.29 (0.36)	0.32 (0.41)	0.39 (0.50)	0.25 (0.18)	0.29 (0.35)	0.87
*Monoglobus*	0.21 (0.38) ^a^	0.96 (1.99)	0.55 (0.53)	0.24 (0.21) ^d^	0.29 (0.72)	**0.02**
*Clostridium sensustricto 1*	0.32 (0.76)	0.37 (0.69)	0.07 (0.053)	0.14 (0.25)	0.28 (0.69)	0.70
*Collinsella*	0.25 (0.67)	0.74 (1.81)	0.20 (0.21)	0.18 (0.19)	0.27 (0.73)	0.45
*Odoribacter*	0.24 (0.22)	0.073 (0.069)	0.30 (0.35)	0.31 (0.52)	0.24 (0.27)	0.34
*Butyricimonas*	0.20 (0.30) ^a^	0.70 (1.37) ^d^	0.35 (0.25)	0.18 (0.20)	0.24 (0.44)	**0.05**
*Bifidobacterium*	0.26 (0.67)	0.11 (0.18)	0.08 (0.084)	0.07 (0.075)	0.22 (0.58)	0.67
*Lachnospiraceae CG 004*	0.22 (0.29)	0.098 (0.14)	0.20 (0.19)	0.30 (0.32)	0.22 (0.28)	0.59
*Eubacterium ruminantium group*	0.20 (0.36)	0.32 (0.67)	0.11 (0.14)	0.23 (0.28)	0.21 (0.36)	0.77
*Megasphaera*	0.20 (0.53)	0.09 (0.11)	0.19 (0.34)	0.28 (0.87)	0.20 (0.54)	0.92
*Eubacterium eligens group*	0.21 (0.37)	0.009 (0.015)	0.165 (0.162)	0.16 (0.13)	0.18 (0.33)	0.56
*Holdemanella*	0.18 (0.29)	0.16 (0.20)	0.18 (0.28)	0.11 (0.16)	0.17 (0.27)	0.91
*Eubacterium xylanophilum group*	0.16 (0.63)	0.027 (0.067)	0.10 (0.07)	0.36 (0.72)	0.18 (0.60)	0.69
*unculturedZ*	0.15 (0.71)	0	0.49 (0.47)	0.08 (0.17)	0.16 (0.64)	0.51
*Lachnoclostridium*	0.15 (0.33)	0.06 (0.10)	0.24 (0.44)	0.21 (0.31)	0.15 (0.32)	0.74
*Lachnospiraceae CG001*	0.19 (0.41)	0.001 (0.002)	0.11 (0.15)	0.06 (0.07)	0.15 (0.37)	0.50
*uncultured5*	0.13 (0.18)	0.11 (0.15)	0.12 (0.10)	0.24 (0.38)	0.14 (0.20)	0.45
*RF39*	0.15 (0.19)	0.05(0.055)	0.22 (0.29)	0.082 (0.10)	0.14 (0.19)	0.29
*Acidaminococcus*	0.14 (0.27)	0.076 (0.07)	0.080 (0.16)	0.060 (0.12)	0.12 (0.25)	0.67
*Romboutsia*	0.10 (0.13) ^a^	0.32 (0.58) ^d^	0.12 (0.11)	0.082 (0.12)	0.12 (0.19)	**0.05**
*Comamonas*	0.09 (0.16) ^c^	0.014 (0.03) ^d^	0.002 (0.005) ^e^	0.45 (1.17)	0.11 (0.41)	**0.04**
*Allisonella*	0.13 (0.22)	0.0488 (0.05)	0.044 (0.05)	0.084 (0.06)	0.11 (0.19)	0.49
*Citrobacter*	0.14 (0.36)	0.036 (0.06)	0.030 (0.07)	0.020 (0.05)	0.01 (0.31)	0.53
*Anaerostipes*	0.11 (0.17)	0.041 (0.06)	0.068 (0.08)	0.12 (0.19)	0.01 (0.16)	0.62
*Parasutterella*	0.12 (0.50)	0.12 (0.31)	0.035 (0.074)	0.03 (0.08)	0.11 (0.45)	0.90
*Lachnospira*	0.10 (0.16)	0.09 (0.11)	0.06 (0.10)	0.12 (0.21)	0.01 (0.15)	0.87
*Prevotellaceae NK3B31 group*	0.11 (0.23)	0.03 (0.04)	0.05 (0.11)	0.01 (0.018)	0.09 (0.21)	0.33
*Erysipelotrichaceae CG003*	0.10 (0.28)	0.02 (0.03)	0.017 (0.015)	0.06 (.09)	0.08 (0.25)	0.72
*Bilophila*	0.07 (0.11) ^a^	0.02 (0.04) ^a^	0.262 (0.269) ^f^	0.03 (0.04) ^d^	0.09 (0.33)	**0.01**
*Eubacterium ventriosum group*	0.07 (0.11)	0.02 (0.04)	0.262 (0.269) ^b^	0.03 (0.04) ^e,f^	0.08 (0.13)	**0.001**
*Unassigned 5*	0.09 (0.22)	0.002 (0.005)	0.044 (0.10)	0.04 (0.08)	0.07 (0.20)	0.65
*Unassigned 6*	0.07 (0.23)	0.06 (0.09)	0.14 (0.21)	0.01 (0.03)	0.07 (0.21)	0.68
*Klebsiella*	0.08 (0.55)	0	0.07 (0.12)	0.06 (0.21)	0.07 (0.48)	0.98
*Slackia*	0.07 (0.21)	0.01 (0.03)	0.020 (0.021)	0.10 (0.28)	0.07 (0.20)	0.77
*Oscillibacter*	0.07 (0.10)	0.047 (0.064)	0.071 (0.083)	0.04 (0.05)	0.69 (0.09)	0.70
*Mitsuokella*	0.04 (0.08)	0.09 (0.10)	0.03 (0.04)	0.11 (0.20)	0.05 (0.10)	0.19
*Weissella*	0.05 (0.15)	0.03 (0.05)	0.01 (0.02)	0.10 (0.24)	0.05 (0.15)	0.71
*Clostridia vaadin BB60 group*	0.05 (0.11)	0.03 (0.07)	0.06 (0.10)	0.05 (0.09)	0.05 (0.10)	0.96
*Prevotellaceae UCG003*	0.02 (0.13) ^a^	0.26 (0.65)	0	0.06 (0.19)^f^	0.04 (0.20)	**0.05**
*Ruminococcus gnavus group*	0.03 (0.10)	0.09 (0.15)	0.13 (0.17) ^b^	0.006 (0.013) ^e^	0.04 (0.11)	0.06
*Unassigned 8*	0.04 (0.15)	0	0.02 (0.023)	0.04 (0.07)	0.04 (1.38)	0.88
*Family XIII UCG 001*	0.03 (0.07)	0.003 (0.008)	0.03 (0.07)	0.04 (0.07)	0.04 (0.07)	0.73
*Unassigned 9*	0.04 (0.16)	0.01 (0.02)	0.003 (0.008)	0.002 (0.006)	0.034 (0.14)	0.78
*Lachnospiraceae UCG003*	0.03 (0.13)	0	0	0.01 (0.03)	0.03 (0.12)	0.71
*Fusobacterium*	0.01 (0.09)	0	0.15 (0.37) ^b^	0.001 (0.004) ^e,f^	0.026 (0.13)	**0.05**
*Uncultured 1*	0.01 (0.04) ^c^	0.009 (0.017)	0.001 (0.002)	0.07 (0.23)	0.019 (0.08)	0.18
*Unassigned 10*	0.02 (0.09)	0	0.004 (0.010)	0.007 (0.022)	0.017 (0.08)	0.84
*Lactobacillus*	0.01 (0.06)	0	0.001 (0.002)	0.02 (0.08)	0.015 (0.06)	0.8
*Anaerovibrio*	0.01 (0.05)	0	0	0.006 (0.020)	0.010 (0.04)	0.81
*Hungatella*	0.008 (0.037)	0	0.03 (0.08)	0.002 (0.009)	0.009 (0.040)	0.37
*Raoultella*	0.004 (0.03) ^c^	0	0	0.03 (0.10)	0.007 (0.043)	0.23
*Tyzzerella*	0.006 (0.019)	0	0.002 (0.005)	0.008 (0.022)	0.005 (0.018)	0.76
*Megamonas*	0.002 (0.01)	0.008 (0.020)	0	0	0.002 (0.013)	0.66
*Treponema*	0.0008 (0.005)	0	0	0.008 (0.03) ^c^	0.001 (0.09)	0.12
*Asteroleplasma*	0.0002 (0.001)	0	0	0	0.001 (0. 001)	0.88
*Paeniclostridium*	0	0	0	0	0	0

^a^: Significant *p*-value obtained from post hoc test of OCG against OD. ^b^: Significant *p*-value obtained from post hoc test of OCG against OAx. ^c^: Significant *p*-value obtained from post hoc test of OCG against ODAx. ^d^: Significant *p*-value obtained from post hoc test of OD against ODAx. ^e^: Significant *p*-value obtained from post hoc test of OAx against ODAx. ^f^: Significant *p*-value obtained from post hoc test of OD against OAx. Text in bold denotes statistical significance
